# hERG1 Potassium Channels: Novel Biomarkers in Human Solid Cancers

**DOI:** 10.1155/2015/896432

**Published:** 2015-08-03

**Authors:** Elena Lastraioli, Tiziano Lottini, Lapo Bencini, Marco Bernini, Annarosa Arcangeli

**Affiliations:** ^1^Experimental and Clinical Medicine, University of Florence, Viale GB Morgagni 50, 50134 Florence, Italy; ^2^General Surgery and Surgical Oncology, Careggi University Hospital, Largo A Brambilla 3, 50134 Florence, Italy; ^3^Breast Unit Surgery, Department of Oncology, Careggi University Hospital, Largo A Brambilla 3, 50134 Florence, Italy

## Abstract

Because of their high incidence and mortality solid cancers are a major health problem worldwide. Although several new biomarkers and potential targets for therapy have been identified through biomolecular research in the last years, the effects on patients' outcome are still unsatisfactory. Increasing evidence indicates that hERG1 potassium channels are overexpressed in human primary cancers of different origin and several associations between hERG1 expression and clinicopathological features and/or outcome are emerging. Aberrant hERG1 expression may be exploited either for early diagnosis (especially in those cancers where it is expressed in the initial steps of tumor progression) or for therapy purposes. Indeed, hERG1 blockage impairs tumor cell growth both *in vitro* and *in vivo* in preclinical mouse model. hERG1-based tumor therapy in humans, however, encounters the major hindrance of the potential cardiotoxicity that many hERG1 blockers exert. In this review we focus on recent advances in translational research in some of the most frequent human solid cancers (breast, endometrium, ovary, pancreas, esophagus, stomach, and colorectum) that have been shown to express hERG1 and that are a major health problem.

## 1. Introduction

A biomarker is defined as a biological molecule indicating atypical processes or disease that can be detected in tissues, blood, and other body fluids. Biomarkers can be used to evaluate the response to a particular treatment. In this view, oncology research greatly relies on biomarkers for diagnostic, prognostic, and predictive purposes.

In recent years, ion channels have been proven to be expressed in different human cancers where they regulate several cancer cell processes. In this view, ion channels could represent novel cancer biomarkers, once properly validated in the clinical setting.

Ion channels are pore-forming transmembrane proteins that regulate passive ion fluxes that are important for key cell processes (i.e., secretion, cell volume regulation). Ion channels are good potential markers because of their localization at the plasma membrane level. This fact makes their detection (e.g., by immunohistochemistry (IHC)) easy and their block with specific drugs and antibodies quick and tunable.

Among ion channels, those encoded by the ether-à-go-go related gene 1 (*hERG1 *also named* KCNH2*) are often overexpressed in neoplastic cell lines and human primary cancers of different histogenesis (reviewed in [1]). hERG1 belongs to an evolutionarily conserved multigenic family of voltage-activated, outward rectifying K^+^ channels, the EAG family. Physiologically, hERG1 channels are responsible for the potassium current (*I*_Kr_) that mediates the rapid repolarizing phase following cardiac action potential.

**Figure 1:**
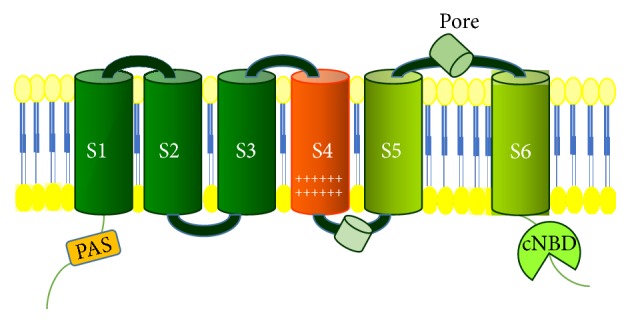
Structure of hERG1 potassium channel. PAS: PAS (acronym of Per Arnt Sim) domain; cNBD (cyclic nucleotide binding domain).

The* KCNH2 *gene (formerly indicated as* hERG1*) was cloned in 1994 from a human hippocampal cDNA library and it is localized on chromosome 7, in q35-36 position [2]. hERG1 channel is composed of 1159 amino acids, and both amino- and carboxy-terminals are located in the cytoplasm (Figure 1).

Functional hERG1 channels are tetramers, and each subunit is made of 6 transmembrane domains (S1–S6) and a long loop which constitutes the pore. Once assembled in the tetramers, the four loops contribute to form the aqueous pore in the center of the structure. The fourth transmembrane segment (S4) contributes to form the voltage sensor domain (VSD), which sharply contributes to hERG1 biophysical properties [3].

In recent years, a progressively more defined picture is emerging, in which hERG1 channels are expressed in several types of human cancers [4] and regulate different cellular processes [5–8]. hERG1 channels are overexpressed in human primary cancers of different histogenesis such as endometrial [9], colorectal [10], esophageal [11], and pancreatic [12] adenocarcinomas and ovarian [13] and brain cancers [14] as well as leukemias [15, 16].

It has been shown that hERG1 is not expressed by the majority of normal nonexcitable tissues as well as hyperplastic lesions (adenomas) [9–11]. Data gathered in the last 15 years underlined that hERG1 channels are important modulators of apoptosis [17] and cell proliferation in leukemias [15, 16] and neuroblastomas [18]. However these tumors will not be discussed in the present review and we refer to a more focused review [19].

Cancers of the breast and reproductive system in females and tumors of the gastrointestinal tract in both sexes collectively represent a major health problem either for their high incidence or poor outcome. Pieces of evidence have been gathered that all the above-mentioned cancer types express high levels of hERG1 channels.  Table 1 summarizes data gathered so far concerning hERG1 expression in cell lines and in solid human cancers.

**Figure 2:**
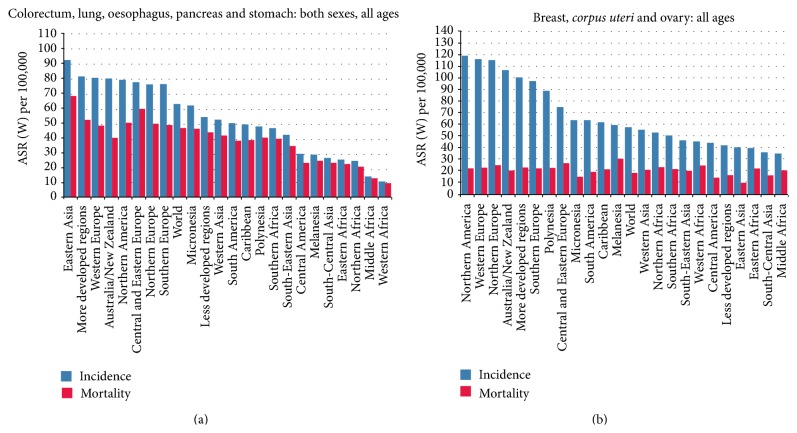
Incidence and mortality for the human solid tumors discussed in the present review. Colorectal, lung, esophageal, pancreatic, and gastric cancers in both sexes (a) and breast,* corpus uteri*, and ovarian cancers in females (b). Source: GLOBOCAN 2012.

From an epidemiologic point of view the above-mentioned solid cancers (breast, endometrium, ovary, esophagus, stomach, colorectum, and pancreas) represent a vast share of both incidence and mortality for cancer worldwide [20] (Figures 2(a) and 2(b)).

Five-year survival rates vary from 89.2% in women affected by breast cancer to 81.5% in women suffering from* corpus uteri* (endometrial) cancer and to 44.6% in women with ovarian cancer; in both sexes, 5-year survival rates vary from 64.7%, 28.3%, 17.5%, 16.8%, and 6.7% in patients with colorectal, gastric, esophageal, lung, and pancreatic cancer, respectively [21, 22].

The differences in survival are mainly represented by distinct biomolecular features as well as efficacy of prevention, diagnostic accuracy, and response to treatment. Nowadays, all these cancers require a multimodal approach that includes oncologists, surgeons, and radiotherapists, although the contribution of many other professionals is often of crucial importance.

The purpose of this paper is to review the recent advances in hERG1 research from cancers arising in breast, female reproductive system, and digestive tract.

## 2. Breast Cancer

Breast cancer (BC) is the most common malignancy among women worldwide and remains the primary cause of death from cancer in females [36]. Unfortunately, BC incidence is increasing everywhere and in less developed countries BC is becoming a major health issue [36–38]. On the other hand, mortality rates for BC are decreasing [36, 39] and it has been estimated that lung cancer instead of BC will become the first cause of death among women and in Europe in 2014, for the first time [39].

A better knowledge of biological features, screening protocols, and access to cutting edge therapies plays a key role in BC treatment. Fisher [40] dramatically changed the perception of BC, introducing the idea of a complex disease from the very beginning of the pathogenetic process, with different factors involved in the natural history of this cancer. Nowadays, not only the TNM stage but also the biological subtypes are crucial for BC clinical management. In order to get a more accurate prognosis and prediction of therapy benefits physicians should use accurate molecular technologies [41]. However, due to the high costs of such techniques, surrogate definitions of subtypes (i.e., hormones expression, proliferation index, and HER-2 expression) obtained through IHC have become a valuable approach for clinicians [42]. The choice of endocrine therapy, chemotherapy regimens, monoclonal antibodies, or kinase inhibitors is mostly driven by the above biomarkers. A striking example of it is the target therapy on HER-2 receptor employing the monoclonal antibody Trastuzumab. Such treatment has significantly changed survival rates in HER-2 positive BC [43]. Hormone-responsive and HER-2 positive cancers are candidate of a specific “biological therapy.” On the contrary, triple negative cases, being devoid of any peculiar biomarker, can only be treated with strong chemotherapy regimens. Moving down this line, identification of biomarkers in BC is of utmost clinical importance either as prognostic tools or as possible therapeutic targets.

Ion channels could therefore represent novel biomarkers in BC. Indeed several studies have already been published addressing the expression of single ion channel types in BC. More recently an ion channel molecular profile was defined for BC, opening interesting perspectives in this field [44].

Long ago it was shown that hERG1 gene is expressed in BC cell lines [4]. More recently, it was shown that hERG1 hyperstimulation in SKBR3 and MDA-MB-231 cells might induce cell senescence [24]. In particular, the authors showed that the exposure to hERG1 channel agonist (NS1643) causes the cell cycle arrest in G0/G1 and induces cell senescence [24].

To our knowledge, no data have been gathered regarding hERG1 channel expression in primary BC so far. Through IHC experiments we demonstrated that hERG1 is overexpressed in primary BC and correlates with clinicopathological parameters such as molecular subtype, grading, ER, and ki67 expression (Lastraioli et al., submitted to Br J Cancer). These findings might be useful in the clinical setting. It is worth recalling here that Tamoxifen (one of the most used drugs for BC treatment) was shown to block hERG1 currents [23], a fact that could explain the QT interval elongation observed in Tamoxifen-treated patients. Due to the high expression in BC and to the sensitivity to Tamoxifen it could be argued that hERG1 might serve as therapeutic targets and/or predictors of response to therapy, although further studies are surely needed.

## 3. Esophageal Cancer

Esophageal cancer represents the sixth leading cause of mortality from cancer worldwide, with a 5-year mortality of less than 15% [20, 22]. The unsatisfactory results are mainly related to late diagnosis and complex therapeutic approaches that include combined regimens combining surgery (with high morbidity and mortality), radiotherapy, and chemotherapy. The current curative algorithm requires perioperative radiochemotherapy and demolitive surgery, although many patients presenting with inoperable locoregional advanced cancers or distant metastatic spread are proposed for palliative radiochemotherapy or support therapy alone [45]. Some new molecular therapies are currently incorporated into classical chemotherapy regimens, but results obtained so far are not satisfactory [46]. From a histopathological point of view, two types of cancer are the most frequent: esophageal squamous-cell carcinoma (ESCC) and esophageal adenocarcinoma (EA), with some differences in geographic prevalence and risk factors [47]. A well-known precursor lesion for EA is Barrett's esophagus (BE). BE is a condition where the normal squamous epithelium of the esophagus is replaced by columnar epithelium of intestinal appearance. BE is currently diagnosed based on the presence of goblet cells of the intestinal type within columnar epithelium [47]. There is currently no evidence that BE screening effectively reduces EA incidence and mortality; nevertheless in 2008 the American Gastroenterological Association Institute recommended performing screening and surveillance in patients with chronic gastroesophageal reflux disease symptoms. Although endoscopic surveillance might lead to early diagnosis, biomarkers would be more useful, as they would allow the measurement of distinct molecular alterations within the tissue. Among these alterations, the best characterized is p53. The immunohistochemical detection of p53 shows correlations with tumor progression and has been validated in big cohorts of patients. The technical easiness of detection makes it a promising biomarker to be translated into clinical practice (reviewed in [48]).

The expression and prognostic role of hERG1 was investigated in ESCC by means of IHC and RT-PCR [26].* KCNH2 *gene and hERG1 protein were found to be expressed in a high percentage of ESCC samples (with respect to normal esophageal squamous epithelium) but no correlations emerged with clinicopathological features. The authors showed that survival rates of hERG1-positive patients were shorter than hERG1-negative patients [26].

A similar approach was applied to the study of EA samples. In particular, the expression of hERG1 protein was detected in BE-derived adenocarcinomas [11].

In 2006, we published the results of a multicentric study [11] showing that hERG1 is overexpressed in the majority of BE samples (69%) while it is absent in normal esophageal mucosa as well as samples taken from patients affected by esophagitis. It was also shown that hERG1 expression is switched on at early stages of BE cancerogenesis and it is also highly expressed in dysplasias and BE-derived adenocarcinomas, thus characterizing both early and late steps of esophageal cancerogenesis [11].

hERG1 channel expression also shows a significant association with malignant progression towards adenocarcinoma, since 89% of BE patients who developed EA were positive for hERG1 protein expression [11]. On the whole, hERG1 channels might identify high-risk BE patients and could therefore be useful for endoscopic surveillance of BE patients in order to ensure a better follow-up and early EA diagnosis.

## 4. Gastric Cancer

Gastric cancer (GC) is the third commonest cause of specific death worldwide and 5-year survival is less than 30% [20, 21]. Many risk factors were investigated, including dietary regimen, smoking habits, alcohol consumption, genetic predisposition, and* Helicobacter pylori* chronic infection [48, 49]. The management of patients without distant metastases is pivoted on surgical resection, although recent clinical trials include perioperative chemotherapy or radiochemotherapy especially in cancer arising from the esophagogastric junction [48]. As many other gastrointestinal cancers, the multimodal management is guided by a correct preoperative TNM stage definition [45]. From a histopathological point of view, about 90% of GCs are classified as adenocarcinomas divided into two subtypes according to Lauren's classification, the intestinal and diffuse type showing different biological and etiological characteristics.

Recently, biomolecular patterns of GC were investigated, including E-cadherin, VEGF, and microsatellite instability [50]. The purpose of these studies was to develop new targeted therapies to improve the poor prognosis achieved by standard chemotherapy. To date, the only clinical trials available are those employing Trastuzumab (with chemotherapy) in HER2-positive advanced GC [51].

hERG1 channels have been proven to be expressed in GC cell lines and primary GCs. In GC cell lines it was shown that hERG1 regulates tumor proliferation [27] and that treatment with hERG1 specific blockers and siRNA impairs tumor growth [29, 52]. hERG1 expression was demonstrated also in primary GCs where it correlates with grading, TNM stage, and serosal and venous invasion [30, 31]. More recently, through an IHC-based study in a wide cohort of GC patients it was demonstrated that hERG1 channels are overexpressed in gastric adenocarcinomas, especially in those of Lauren intestinal type [31]. hERG1 expression also correlated with grading, TNM stage, and VEGF-A expression. Moreover, in GC cell lines it was shown that hERG1 modulates VEGF-A secretion through an AKT-dependent pathway [31]. Even more interestingly, by treating xenografted cancers with a combination of hERG1 blockers and Bevacizumab the effect was greater than that obtained with single-agent treatment [31].

Overall, data gathered so far are still contradicting, since Ding and colleagues proposed hERG1 as an independent prognostic factor [30] while in our series hERG1 identifies high-risk T1 patients [31] but is not an independent prognostic factor. It should be pointed out that the cohort analyzed in our study was bigger than the one analyzed by Ding and colleagues and it was composed of Italian patients, who display different characteristics than Asian patients. Moreover, a different antibody and scoring method was applied and this could account for differences in the obtained results.

In primary GC it was also demonstrated that hERG1 channels are expressed in the early stages of the disease (manuscript in preparation and [52]). By means of IHC we showed that hERG1 channels are expressed from the early steps of GC progression (gastric metaplasia) and that such expression is maintained during all the phases of the cancerogenic process [52].

Overall, the detection of hERG1 expression in gastric dysplastic lesions could therefore represent a novel prognostic marker of progression towards gastric adenocarcinoma of the intestinal histotype.

## 5. Colorectal Cancer

Colorectal cancer (CRC) is the fourth most common cause of death for cancer worldwide, with a 5-year survival rate higher than 60% taking into account CRC encompassing all the pathological stages [20, 21]. The prognosis of CRC patients has been consistently improving during the last decades due to many important developments in prevention, early diagnosis, and therapy. For example, the widespread screening colonoscopy has led to reduced cancer incidence (for benign or preneoplastic adenomas removal) and mortality (due to early diagnosis) [53]. TNM staging system is highly correlated with prognosis, with a 5-year survival of 90% for patients in earlier stages to less than 25% for those with metastatic disease [53]. The cornerstone of therapy is represented by en bloc surgical resection of tumor and regional nodes, although perioperative chemotherapy is mandatory in subjects with advanced disease and metastasis.

The most frequent histological subtype is adenocarcinoma, accounting for more than 95% of the cases and the molecular pathogenesis of colorectal cancer has been widely studied. The molecular sequence from adenoma to invasive cancer is well established, with the identification of misexpression and mutation of several genes (including rare inherited syndromes). Many molecular targets are currently used for prognostic and predictive purposes. In particular* k-ras* mutation profile is used to refine prognosis and to select patients who will benefit from treatment with anti-EGF-R antibodies. For therapy purposes, anti-VEGF-A antibodies have been employed in addition to standard chemotherapy agents. Despite all the efforts, the prognosis of patients with advanced stage disease has not been significantly improved [54, 55].

hERG1 protein is highly expressed in colorectal adenocarcinomas with respect to hyperplastic lesions of the colon [11] and in CRC cell lines [11, 33] and it was demonstrated that the protein is not expressed in small adenomas and sigma diverticulitis [56]. In CRC cell lines, a correlation between invasive phenotype and high hERG1 levels of expression has been shown [11] and proliferation assays demonstrated that treating the cells with a specific hERG1 blocker (E4031) reduced tumor growth [56]. The effects of a different hERG1 blocker (sparfloxacin, SPFX) were tested on colon cancer cells with a high hERG1 expression [57]. The authors showed that SPFX inhibits cell proliferation, migration, and apoptosis and a synergistic effect was observed treating the cells in combination with 5-FU [57].

In CRC cell lines it was also demonstrated that hERG1 channels modulate tumor progression by switching on a VEGF-A-dependent angiogenic pathway [33]. hERG1 expression was also evaluated in mouse models [58]. It was shown that colonic polyps of adenomatous polyposis coli (Apc^min⁡/+^) mice expressed the murine homolog of hERG1 (mERG1) and that treating the animals with a specific hERG1 blocker reverted polyps development [58]. Treating transgenic mice (overexpressing hERG1 in the colorectal mucosa) with a chemical carcinogen (Azoxymethane) resulted in an increased number of mucin-depleted foci and polyps [58].

Finally, in a cohort of primary nonmetastatic CRC samples it was shown that hERG1 expression was associated with Glut-1 (glucose transporter 1), VEGF-A, CA-IX (carbonic anhydrase IX), and EGF-R expression [34]. In a multivariate model, TNM, hERG1, and Glut-1 turned out to be prognostic factors [34]. Moreover, hERG1 presence associated with Glut-1 absence represents an independent negative prognostic factor in TNM I and II colorectal adenocarcinomas [34].

On the whole, data gathered so far in CRC cell lines, primary CRCs, and mouse models indicate that hERG1 has a role in CRC cancerogenesis that can be traced back to the regulation of VEGF-A signaling pathway [33, 34, 59]. Moreover, hERG1 has a prognostic value in CRC [47] and all these data stress the necessity of including hERG1 blocking therapeutic strategies in CRC treatment schedules.

## 6. Pancreatic Cancer

Pancreatic cancer (PC) is responsible for 6.8% of all cancer-related deaths [22]. PC incidence and mortality have been steady in the last 20 years, and despite recent efforts to optimize treatments, 5-year survival rate is still poor (6.7%) [22]. Several risk factors for PC have been described. 20% of PC is likely to be induced by cigarette smoking [21]. It has been shown that a family history of PC also increases the risk of developing PC [59] as well as a personal history of chronic pancreatitis, obesity, and diabetes [21]. From a histopathological point of view, 90% of PC is classified as ductal adenocarcinomas (PDAC), with the other histotypes accounting all together for the remaining 10%.

Unfortunately, currently there are no screening detection methods and the vast majority of PC is diagnosed when the disease has already spread beyond the pancreas. For these reasons, surgery and radiochemotherapy are used as treatment options but they can be curative only in a small percentage of patients. For PC patients presenting with advanced disease, chemotherapy with Erlotinib plus gemcitabine has been used, with a slight survival improvement.

Although recently several studies have been performed aimed at identifying novel prognostic and predictive molecular biomarkers, none of them can be included in routine clinical use yet [60].

Recently, it was demonstrated that hERG1 channels are highly expressed in primary PC and PC cell lines [12]. In PC samples, hERG1 expression was correlated with lymph node involvement, grading, and TNM stage I [12]. The authors also showed that hERG1 is a target of miR-96 (microRNA-96, which is downregulated in PC) and that miR-96 overexpression regulates hERG1 expression hence significantly inhibiting PC cells malignant behavior.

We recently showed that hERG1 channels are overexpressed in human PC samples of the ductal type (PDAC) and correlate with EGF-R [61]. Moreover, blocking hERG1 in PDAC cells reduces cell growth and migration and we demonstrated that PDAC patients with high hERG1 expression had a worse prognosis.

## 7. Other Cancers

### 7.1. Endometrial Cancer

Endometrial cancer (EC) is nowadays the most common gynecologic malignancy and the most frequent among infiltrating tumors of the female genital tract, especially after menopause. About 75% of the cases affect* corpus uteri* and 15–20% of these show relapse and do not respond to systemic therapy [62]. Approximately 70 to 80% of EC patients have a localized disease that is treated by surgery alone; nevertheless roughly 30% of the patients will die from the disease. In this scenario, it appears clear that EC is a heterogeneous disease and novel biomarkers are urgently needed. This will allow better stratifying EC patients and ensuring the best treatment options. Biomolecular research has identified new targets for EC therapy such as mTOR (mammalian target of rapamycin, in particular for type I EC), p53 and HER-2 (human epidermal growth factor receptor 2, especially in type II EC), VEGF (vascular endothelial growth factor), and EGF-R (epidermal growth factor receptor) (reviewed in [63]). A recent report summarized data concerning new molecular markers in EC pointing out the relevance of several markers (p53, aneuploidy, HER-2, estrogen receptors, progesterone receptors, and Stathmin) [64].

The first paper demonstrating the expression of hERG1 potassium channels in human primary cancers was conducted on EC samples [9]. In such paper it was demonstrated that* hERG1* mRNA can be detected in human tissues by end-point RT-PCR (Retrotranscription-Polymerase Chain Reaction) as well as by IHC and is more frequently expressed in human neoplastic tissues compared to normal endometrium and hyperplastic lesions [9]. Furthermore, patch clamp analysis indicated that functional hERG1 proteins are expressed on the cell surface of EC cells. This paper opened the way for further investigation of hERG1 expression in clinical samples, although the analysis was carried out on a small group of EC patients.

### 7.2. Ovarian Cancer

Ovarian cancer (OC) represents the leading cause of death among gynecologic malignancies, despite recent efforts in surgical and chemotherapy treatments. Currently, 5-year survival for OC is 44.6% [22]. The gold standard for OC treatment is surgery usually followed by chemotherapy [21]. Currently, there is no screening test for OC early detection although pelvic examination associated with transvaginal ultrasound and CA-125 (Cancer Antigen 125) evaluation in blood samples can be proposed to high-risk women, in particular those who have a family history positive for BC and OC.

Several molecules have been proposed as tumor markers for OC. The most used serum marker is CA-125 but many others have been tested as well (such as HE4 (human epididymal protein E4), Kallikreins, Osteopontin, Claudins, and VCAM-1 (vascular cell adhesion molecule 1)) (reviewed by [65]). Among epigenetic aberrations that might be used as biomarkers, the best characterized is* BRCA1* hypermethylation which leads to the absence of mRNA and protein and correlates with poor outcome [66]. Since VEGF-A and VEGFR-2 (vascular endothelial growth factor receptor 2) expression might be associated with reduced survival, a phase III clinical trial employing Bevacizumab in recurrent platinum-resistant OC patients (AURELIA Trial) was designed and such treatment resulted in a significant improvement of PFS (progression-free survival) and ORR (objective response rate) [67].

On the whole, despite the wide spectrum of serum biomarkers identified, unsatisfactory clinical results have been achieved. For these reasons, search for new biomarkers is mandatory.

A few studies have been performed to evaluate hERG1 expression and role in OC. A paper published in 2010 [13] demonstrated that hERG1 is expressed in OC cell lines and primary samples but no associations with survival emerged. More recently, a methylation profile for clear-cell OC was defined [25]. Among the nine genes investigated the authors included hERG1 potassium channel and they showed that the gene was methylated and hence its expression in the tumor tissue was lower, indicating epigenetic silencing. Those results, although obtained in a small set of OC samples, might indicate that that loss of hERG1 expression by methylation could represent a potential prognostic marker.

## 8. Concluding Remarks

Despite the improvements in surgical techniques and chemotherapy schedules, the treatment of solid cancers is still a big challenge for surgeons and oncologists. Therefore, targeted therapy represents the best opportunity for the treatment of patients not responding to classical approaches. Data gathered so far suggest that hERG1 channels could be used as biomarkers since they are frequently overexpressed in solid cancers and such expression associates with clinicopathological features in different tumors. A reliable monoclonal antibody for hERG1 protein is available and evaluation scores have been optimized in different solid cancer, thus making the detection of the channel easy for pathologists. Moreover, being a transmembrane protein, hERG1 is easily accessible and might be targeted by several small molecules that might be associated with the treatment with drugs already used in the clinical settings, contributing to lower costs of cancer patients' treatment. Moreover, anti-hERG1 antibody and its derivative scFv (Single Chain Variable Fragment) might be conjugated with drugs for treatment. Different strategies might be applied such as targeting specific conformations of hERG1 channels and using new molecular tools aimed at decreasing hERG1 expression in tumor cells only to decrease channel expression in selected cancer types (for a more detailed review see [19, 68]).

## Figures and Tables

**Table 1:** hERG1 expression and role in cell lines and in human solid tumors.

Tumor type	Cell lines	Human tumors
Breast cancer	hERG1 current is blocked by Tamoxifen [23]; induction of cell senescence [24]	—

Endometrial cancer	—	Overexpression [9]

Ovarian cancer	Expression [13]	Overexpression [13]; methylation and downregulation [25]

Esophageal cancer	—	Overexpression in EA and BE [11], ESCC [26]; malignant progression [11]

Gastric cancer	Cell proliferation [27]; apoptosis [28]	Grading, TNM stage, serosal, and venous invasion [29, 30]; Lauren's intestinal type, localization (fundus), low grading, and early stages (TNM I and II) [31]; in early stage (T1) HERG1 expression identified high-risk patients [31]

Colorectal cancer	Invasiveness [10]; chemosensitivity [32]; regulation of VEGF-A secretion [33]	++, correlation with invasive phenotype [10]; independent negative prognostic factor in stage I and II CRC [34]

Pancreatic cancer	Overexpression [12]	Lymphnode involvement, grading, and TNM stage I [12]

Lung cancer	Cell proliferation [35]	—

EA: esophageal adenocarcinoma; BE: Barrett's esophagus; ESCC: esophageal squamous cell carcinoma; TNM: tumor node metastasis; VEGF-A:vascular endothelial growth factor.
